# TTR: Time in Therapeutic Range or “The Troublesome Report”?

**DOI:** 10.19102/icrm.2019.100103

**Published:** 2019-01-15

**Authors:** James A. Reiffel

**Affiliations:** ^1^Electrophysiology Section, Division of Cardiology, Department of Medicine, Columbia University, New York, NY, USA

**Keywords:** Anticoagulation, coagulation tests, international normalized ratio, prothrombin time

In the December 2018 issue of the *Journal of Innovations in Cardiac Rhythm Management*, Saddiqui et al.^1^ present an interesting discussion regarding comparative approaches in calculating the time in therapeutic range (TTR) measurement, which is the most common metric used to represent the quality of oral anticoagulation (OAC) with vitamin K antagonists (VKAs) such as warfarin. They reported, through the completion of a 21-week retrospective analysis, that the three different methods generally employed in clinical practice and clinical research—specifically, the traditional, cross-sectional, and Rosendaal methods—do not accurately measure whether a patient is in or out of the therapeutic range because there can be inconsistency between their findings for the same patient over the same time period. The investigators also noted that the addition of tolerances [ie, small widening of the range by 0.2 and 0.5 at either end beyond the standard 2.0 to 3.0 international normalized ratio (INR)] that has been suggested can further distort the perception of anticoagulation achieved. Moreover, Saddiqui et al.^1^ recommended that a standardized TTR calculation method as well as a uniform tolerance for use in clinical trials and quality control efforts be implemented.

I agree with the authors that TTR is a troublesome report despite its frequent usage. However, beyond the method-related variability in the TTR value obtained and the consequences these differences may have in the context of physician decisions regarding OAC dosing, additional concerns abound, even if we limit our use to just one method such as the Rosendaal approach or a modification thereof, as most recent clinical trials have done. As I have previously reported, both in the pages of this journal and others,^2–4^ just knowing the percentage of time that the INR is in the so-called therapeutic range of 2.0 to 3.0 (ie, the TTR), regardless of the method used for its calculation, does not actually tell the story well enough to truly optimize VKA therapy management.

The now widespread use of TTR is based upon documentation that, as the INR rises above 3.0 (and especially above 3.5 to 4.0), the risk of bleeding on an VKA increases progressively, whereas, as the TTR falls below 2.0, inefficacy with addressing the risk of thromboembolism increases progressively in patients with thromboembolic risk for which the VKA is being given. Accordingly, the more the INR is kept in the “sweet spot” of between 2.0 and 3.0—the therapeutic range—the more the risk of both thromboembolism from inefficacy and bleeding from an excess anticoagulant effect can be kept at a minimum. Thus, the higher the TTR, the more ideal the control of OAC is. It is troublesome, then, that the TTR value so calculated can be different depending upon the method used.

Furthermore, perhaps even more troublesome than the above is the fact that the TTR can be misleading to the user, even when its value is considered high—the latter, as Siddiqui et al.^1^ note, being 50% to 60% in clinical practice or 60% to 69% in clinical research trials. Consider the following: two patients can each have a TTR value of 60% (obtained using the same method to calculate over the same period of time using the same number of measurements). However, in one, the 40% time out of range includes all low INR values (let us say < 1.5), whereas, in the other, the 40% of time out of range includes all high INR values (let us say > 4.0). In the former, the concern would be excess thromboembolism risk, whereas, in the latter, the concern would be excess bleeding risk. Thus, without providing the pattern of those INR values that are out of the therapeutic range, identical TTR values may be quite misleading. When reporting out of range values in addition to the TTR, better corrective actions may be taken and risks minimized.

Importantly, Siddiqui et al. noted that I have previously raised this issue when they said that “it has been suggested that incorporation of more variables in the calculation of TTR, such as in the TTR-F formula proposed by Reiffel, would improve its accuracy as a quality control measure.” However, they then stated that “the level of detail needed (such as mean INR, number of INR measurements, and percentage of INR out of range) raises concern for complexity and time consumption.” Nonetheless, although I agree that any additional measurements one has to gather in order to report a calculation will take additional effort, given the importance of optimizing anticoagulation efficacy and safety, the recognition of the limitations of a test such as the TTR and the making of efforts to improve upon them would be worthwhile for the patients in whom such OAC management is crucial. Interesting in this respect is an email I received from Fritz Rosendaal himself in June 2017 after he read my editorial in *Circulation*,^3^ in which he stated “I never fully understood why such an absolute meaning has been given to the measure [TTR], which as I am sure you know from our 1993 publication, was not developed for this reason [as a uniform measure of OAC management quality].”

Thus, although I agree with the conclusion stated in the Siddiqui et al.^1^ article that they “recommend a standardized TTR calculation method as well as a uniform tolerance for use in clinical trials and quality control efforts [be developed],” I believe that not only should just one approach to the TTR calculation be used if possible, such as that of Rosendaal et al.,^5^ but also that additional clinically important information that was used to contribute to the TTR calculation—such as the specifics of the INR values out of range, the number of INR values utilized, and the median INR value present—be provided.
